# Traumatic Brain Injury–Related Deaths by Race/Ethnicity, Sex, Intent, and Mechanism of Injury — United States, 2000–2017

**DOI:** 10.15585/mmwr.mm6846a2

**Published:** 2019-11-22

**Authors:** Jill Daugherty, Dana Waltzman, Kelly Sarmiento, Likang Xu

**Affiliations:** 1 Division of Injury Prevention, National Center for Injury Prevention and Control, CDC.

Traumatic brain injury (TBI) affects the lives of millions of Americans each year (*1*). To describe the trends in TBI-related deaths among different racial/ethnic groups and by sex, CDC analyzed death data from the National Vital Statistics System (NVSS) over an 18-year period (2000–2017). Injuries were also categorized by intent, and unintentional injuries were further categorized by mechanism of injury. In 2017, TBI contributed to 61,131 deaths in the United States, representing 2.2% of approximately 2.8 million deaths that year. From 2015 to 2017, 44% of TBI-related deaths were categorized as intentional injuries (i.e., homicides or suicides). The leading category of TBI-related death varied over time and by race/ethnicity. For example, during the last 10 years of the study period, suicide surpassed unintentional motor vehicle crashes as the leading category of TBI-related death. This shift was in part driven by a 32% increase in TBI-related suicide deaths among non-Hispanic whites. Firearm injury was the underlying mechanism of injury in nearly all (97%) TBI-related suicides among all groups. An analysis of TBI-related death rates by sex and race/ethnicity found that TBI-related deaths were significantly higher among males and persons who were American Indians/Alaska Natives (AI/ANs) than among all other groups across all years. Other leading categories of TBI-related deaths included unintentional motor vehicle crashes, unintentional falls, and homicide. Understanding the leading contributors to TBI-related death and identifying groups at increased risk is important in preventing this injury. Broader implementation of evidence-based TBI prevention efforts for the leading categories of injury, such as those aimed at stemming the significant increase in TBI-related deaths from suicide, are warranted.

Data from CDC’s NVSS multiple-cause-of-death files were analyzed for 2000–2017. NVSS collects data for all deaths among U.S. residents. TBI-related deaths were classified using codes from the *International Classification of Diseases, Tenth Revision* (ICD-10) using an established surveillance definition (*2*). Deaths were classified as TBI-related if any multiple codes for causes of deaths listed in the death record indicated a TBI-related diagnosis, and the single underlying cause of death was listed as an injury. This methodology represents a change in the calculation of estimates from previous CDC reports (*1*,*2*), which did not require that an injury be listed as an underlying cause of death.^†^ Data on TBI-related deaths were stratified by year, race/ethnicity, sex, and principal mechanism of injury. Racial/ethnic groups included non-Hispanic white (white), non-Hispanic black (black), non-Hispanic American Indian/Alaska Native (AI/AN), non-Hispanic Asian/Pacific Islander (Asian/PI), Hispanic, and other. Injuries were categorized first by intent (intentional, unintentional, and undetermined intent). Intentional injuries were further categorized as suicide or homicide. Unintentional injuries were further categorized by mechanism of injury (motor vehicle crashes, falls, being struck by or against an object, or unspecified). Principal mechanism of injury was categorized based on the CDC-recommended external cause of injury mortality matrix for ICD-10 (*3*) and are presented as the pooled average of 3-year groupings.

Each rate and its corresponding 95% confidence interval were based on U.S. bridged-race population estimates of the resident population (*4*). U.S. census population estimates for the year 2000 were used as the standard for age-adjusted rates by direct method (*5*). T-tests were used to analyze between-group differences for rates of TBI-related deaths. Only selected comparisons were tested for statistical significance. Differences with p-values <0.05 were considered statistically significant. JoinPoint regression software (version 4.7.0.0; National Cancer Institute) was used to calculate the average annual percent changes of TBI-related death rates from 2000 to 2017 for each race and Hispanic origin group to illustrate trends over time. Average annual percent changes were considered significantly different from zero for p-values <0.05. SAS (version 9.4; SAS Institute, Inc.) was used for all statistical analyses.

The overall rate of TBI-related deaths remained constant from 2000 to 2005, followed by a statistically significant decrease in the overall rate from 2005 to 2010 and then a flattening out from 2010 to 2014. From 2014 to 2017, a small but statistically significant increase in the overall rate of TBI-related deaths occurred (Figure). TBI-related death rates were significantly higher among males of all races than among females throughout the study period (p<0.001) (Table 1), and age-adjusted rates were significantly higher among AI/AN persons than among other racial/ethnic groups (p<0.001). From 2001 to 2006, the death rates of whites and blacks were similar (p>0.05), but since 2007, the rate of TBI-related deaths has been significantly higher among whites (p<0.001).

**FIGURE:**
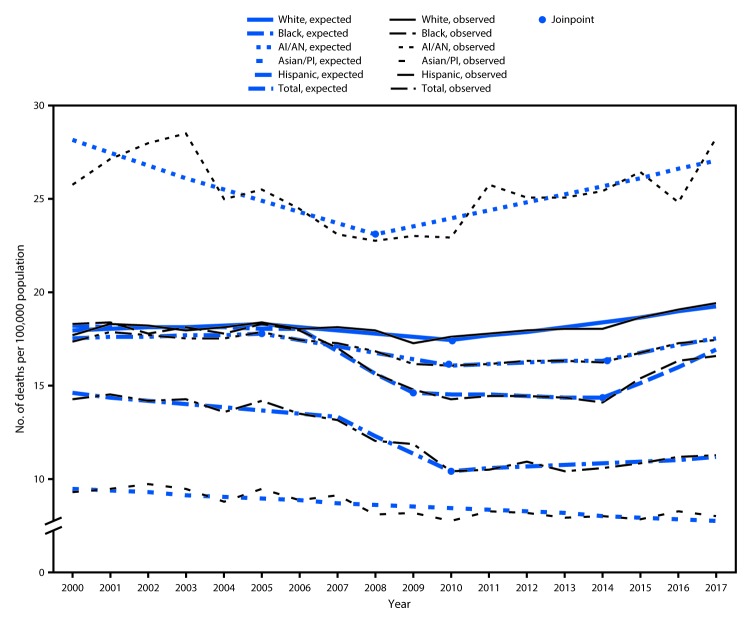
Age-adjusted rates* of traumatic brain injury–related deaths, by year and race/ethnicity^†^ — United States, 2000–2017 **Abbreviations:** AI/AN = American Indian/Alaska Native; A/PI = Asian/Pacific Islander. * Per 100,000 population. ^†^ Whites, Blacks, AI/AN, A/PI were non-Hispanic; Hispanic persons could be of any race.

**TABLE 1:** Estimated number* and age-adjusted rates^†^ of traumatic brain injury (TBI)–related deaths,^§^ by year, sex, and race/ethnicity^¶^ — United States, 2000–2017**

Year/Sex	Race/Ethnicity											Total	
	White		Black		American Indian/Alaska Native		Asian/Pacific Islander		Hispanic		Other		
	No.	Rate (95% CI)	No.	Rate (95% CI)	No.	Rate (95% CI)	No.	Rate (95% CI)	No.	Rate (95% CI)	No.	No.	Rate (95% CI)
**2000**													
Male	26,497	27.6 (27.3–28.0)	4,832	30.5 (29.6–31.4)	412	38.5 (34.3–42.6)	599	13.0 (11.9–14.2)	3,593	22.5 (21.6–23.4)	164	36,097	27.3 (27.0–27.6)
Female	9,982	9.0 (8.8–9.2)	1,436	8.0 (7.5–8.4)	169	14.4 (12.2–16.7)	291	6.0 (5.3–6.7)	912	6.3 (5.8–6.8)	40	12,830	8.5 (8.4–8.7)
Total	36,479	17.7 (17.5–17.9)	6,268	18.3 (17.8–18.8)	581	25.8 (23.6–28.1)	890	9.3 (8.6–9.9)	4,505	14.3 (13.8–14.8)	204	48,927	17.4 (17.2–17.5)
**2001**													
Male	27,747	28.6 (28.3–29.0)	4,915	30.8 (29.9–31.7)	429	39.4 (35.2–43.6)	648	13.8 (12.6–15.0)	3,865	22.7 (21.8–23.6)	166	37,770	28.2 (28.0–28.5)
Female	10,307	9.1 (8.9–9.3)	1,410	7.9 (7.4–8.3)	184	16.0 (13.5–18.4)	306	5.9 (5.2–6.6)	961	6.4 (6.0–6.9)	43	13,211	8.7 (8.5–8.8)
Total	38,054	18.3 (18.1–18.5)	6,325	18.4 (17.9–18.8)	613	27.2 (24.9–29.5)	954	9.5 (8.8–10.1)	4,826	14.5 (14.0–15.0)	209	50,981	17.9 (17.7–18.0)
**2002**													
Male	27,771	28.4 (28.1–28.7)	4,811	30.0 (29.1–30.9)	480	42.2 (38.1–46.4)	652	13.6 (12.4–14.7)	3,908	22.5 (21.6–23.4)	186	37,808	27.9 (27.6–28.2)
Female	10,400	9.1 (9.0–9.3)	1,402	7.6 (7.2–8.0)	171	14.7 (12.4–17.0)	334	6.4 (5.7–7.2)	973	6.1 (5.7–6.5)	31	13,311	8.6 (8.5–8.8)
Total	38,171	18.2 (18.0–18.4)	6,213	17.8 (17.3–18.2)	651	28.0 (25.7–30.3)	986	9.7 (9.1–10.4)	4,881	14.2 (13.7–14.6)	217	51,119	17.7 (17.6–17.9)
**2003**													
Male	27,631	28.0 (27.7–28.4)	4,923	30.0 (29.1–30.9)	491	44.1 (39.8–48.4)	671	13.3 (12.2–14.4)	3,977	22.1 (21.3–23.0)	119	37,812	27.6 (27.3–27.9)
Female	10,439	9.0 (8.8–9.2)	1,472	8.0 (7.6–8.4)	162	14.2 (11.9–16.5)	338	6.1 (5.4–6.8)	1,038	6.4 (6.0–6.9)	40	13,489	8.6 (8.5–8.8)
Total	38,070	18.0 (17.8–18.2)	6,395	18.1 (17.7–18.6)	653	28.6 (26.2–30.9)	1,009	9.4 (8.8–10.0)	5,015	14.2 (13.8–14.7)	159	51,301	17.6 (17.4–17.7)
**2004**													
Male	27,799	27.9 (27.6–28.3)	4,842	29.7 (28.8–30.6)	435	37.9 (34.0–41.8)	604	11.7 (10.7–12.7)	3,938	20.9 (20.1–21.7)	117	37,735	27.2 (26.9–27.5)
Female	10,921	9.4 (9.2–9.6)	1,466	7.9 (7.5–8.3)	163	13.2 (11.1–15.3)	366	6.2 (5.6–6.9)	1,012	6.1 (5.7–6.5)	40	13,968	8.8 (8.7–9.0)
Total	38,720	18.1 (18.0–18.3)	6,308	17.8 (17.4–18.3)	598	25.0 (22.9–27.1)	970	8.7 (8.1–9.3)	4,950	13.5 (13.1–14.0)	157	51,703	17.5 (17.3–17.6)
**2005**													
Male	28,771	28.6 (28.3–29.0)	5,126	30.5 (29.6–31.4)	471	39.0 (35.2–42.7)	741	13.9 (12.9–15.0)	4,261	22.4 (21.5–23.2)	122	39,492	28.0 (27.8–28.3)
Female	10,852	9.2 (9.0–9.4)	1,462	7.9 (7.5–8.3)	157	12.9 (10.8–15.0)	352	5.8 (5.2–6.4)	1,068	6.1 (5.7–6.5)	25	13,916	8.6 (8.5–8.8)
Total	39,623	18.4 (18.2–18.6)	6,588	18.3 (17.9–18.8)	628	25.6 (23.5–27.6)	1,093	9.5 (8.9–10.1)	5,329	14.2 (13.8–14.6)	147	53,408	17.8 (17.7–18.0)
**2006**													
Male	28,336	27.9 (27.5–28.2)	5,205	30.4 (29.5–31.3)	453	37.8 (34.1–41.5)	703	12.6 (11.7–13.6)	4,254	21.3 (20.5–22.1)	105	39,056	27.3 (27.0–27.6)
Female	10,905	9.2 (9.0–9.3)	1,401	7.4 (7.0–7.7)	152	12.1 (10.1–14.0)	355	5.7 (5.1–6.3)	1,025	5.7 (5.3–6.1)	29	13,867	8.5 (8.4–8.6)
Total	39,241	18.0 (17.9–18.2)	6,606	18.0 (17.5–18.4)	605	24.5 (22.4–26.5)	1,058	8.9 (8.3–9.4)	5,279	13.5 (13.1–13.9)	134	52,923	17.4 (17.3–17.6)
**2007**													
Male	28,849	28.1 (27.8–28.4)	4,980	28.2 (27.4–29.0)	422	35.6 (31.9–39.2)	752	12.9 (11.9–13.9)	4,141	20.6 (19.9–21.4)	104	39,248	27.1 (26.8–27.4)
Female	11,003	9.1 (8.9–9.2)	1,395	7.2 (6.8–7.6)	141	11.4 (9.4–13.3)	373	5.8 (5.2–6.4)	1,056	5.7 (5.3–6.1)	29	13,997	8.4 (8.3–8.6)
Total	39,852	18.1 (17.9–18.3)	6,375	17.0 (16.6–17.4)	563	23.1 (21.1–25.1)	1,125	9.1 (8.5–9.6)	5,197	13.1 (12.7–13.5)	133	53,245	17.3 (17.2–17.5)
**2008**													
Male	29,211	28.1 (27.8–28.5)	4,670	26.4 (25.6–27.2)	430	35.2 (31.7–38.8)	703	11.9 (11.0–12.8)	3,810	19.0 (18.3–19.7)	84	38,908	26.5 (26.3–26.8)
Female	10,807	8.7 (8.6–8.9)	1,253	6.4 (6.0–6.7)	139	11.1 (9.2–13.0)	327	4.8 (4.3–5.4)	968	5.2 (4.8–5.6)	32	13,526	8.0 (7.8–8.1)
Total	40,018	18.0 (17.8–18.2)	5,923	15.7 (15.3–16.1)	569	22.7 (20.8–24.7)	1,030	8.1 (7.6–8.6)	4,778	12 (11.6–12.4)	116	52,434	16.8 (16.7–17.0)
**2009**													
Male	28,236	26.9 (26.6–27.2)	4,346	24.5 (23.7–25.3)	411	33.2 (29.8–36.5)	711	11.6 (10.7–12.5)	3,789	18.3 (17.6–18.9)	154	37,647	25.4 (25.1–25.6)
Female	10,610	8.5 (8.3–8.6)	1,298	6.5 (6.1–6.9)	169	13.2 (11.2–15.3)	362	5.2 (4.7–5.7)	1,034	5.4 (5.1–5.8)	43	13,516	7.9 (7.7–8.0)
Total	38,846	17.2 (17.1–17.4)	5,644	14.8 (14.4–15.2)	580	23.0 (21.1–25.0)	1,073	8.1 (7.6–8.6)	4,823	11.8 (11.4–12.2)	197	51,163	16.2 (16.0–16.3)
**2010**													
Male	28,678	27.5 (27.2–27.8)	4,303	24.0 (23.2–24.7)	401	34.4 (30.8–38.1)	749	11.8 (10.9–12.7)	3,381	16.3 (15.6–16.9)	144	37,656	25.3 (25.0–25.5)
Female	10,948	8.7 (8.5–8.8)	1,168	5.8 (5.5–6.2)	150	12.5 (10.4–14.5)	327	4.4 (3.9–4.9)	975	4.9 (4.5–5.2)	40	13,608	7.8 (7.6–7.9)
Total	39,626	17.6 (17.4–17.8)	5,471	14.2 (13.9–14.6)	551	22.9 (20.9–24.9)	1,076	7.7 (7.2–8.2)	4,356	10.4 (10.0–10.7)	184	51,264	16.0 (15.9–16.2)
**2011**													
Male	29,067	27.6 (27.3–27.9)	4,420	24.3 (23.5–25.0)	462	39.3 (35.4–43.1)	798	12.4 (11.4–13.3)	3,581	16.8 (16.1–17.4)	114	38,442	25.4 (25.2–25.7)
Female	11,086	8.8 (8.6–8.9)	1,237	6.0 (5.7–6.4)	166	13.3 (11.2–15.4)	384	5.1 (4.5–5.6)	937	4.6 (4.3–4.9)	36	13,846	7.8 (7.7–7.9)
Total	40,153	17.8 (17.6–17.9)	5,657	14.4 (14.1–14.8)	628	25.7 (23.6–27.9)	1,182	8.3 (7.8–8.8)	4,518	10.5 (10.1–10.8)	150	52,288	16.2 (16.0–16.3)
**2012**													
Male	29,678	27.9 (27.6–28.2)	4,549	24.5 (23.7–25.2)	495	40.3 (36.5–44.0)	797	11.7 (10.8–12.5)	3,700	17.3 (16.6–17.9)	137	39,356	25.7 (25.4–25.9)
Female	11,402	8.9 (8.8–9.1)	1,187	5.7 (5.4–6.0)	144	11.1 (9.2–12.9)	422	5.3 (4.8–5.8)	1,045	5.0 (4.7–5.3)	38	14,238	7.9 (7.8–8.1)
Total	41,080	18.0 (17.8–18.2)	5,736	14.4 (14.0–14.8)	639	25.1 (23.1–27.1)	1,219	8.1 (7.6–8.6)	4,745	10.9 (10.5–11.2)	175	53,594	16.3 (16.2–16.5)
**2013**													
Male	30,118	28.0 (27.7–28.4)	4,525	24.0 (23.3–24.8)	461	38.7 (34.9–42.4)	841	11.5 (10.7–12.3)	3,605	16.4 (15.8–17.0)	120	39,670	25.5 (25.3–25.8)
Female	11,588	9.0 (8.8–9.1)	1,257	5.9 (5.6–6.3)	160	12.7 (10.6–14.7)	424	5.0 (4.5–5.5)	1,044	4.8 (4.5–5.1)	35	14,508	7.9 (7.8–8.1)
Total	41,706	18.1 (17.9–18.3)	5,782	14.4 (14.0–14.7)	621	25.1 (23.1–27.2)	1,265	7.9 (7.5–8.4)	4,649	10.4 (10.0–10.7)	155	54,178	16.3 (16.1–16.4)
**2014**													
Male	30,432	28.0 (27.6–28.3)	4,501	23.6 (22.8–24.3)	486	39.6 (35.9–43.4)	888	11.6 (10.8–12.4)	3,738	16.5 (15.9–17.1)	152	40,197	25.5 (25.2–25.7)
Female	11,714	9.0 (8.8–9.2)	1,242	5.9 (5.6–6.2)	158	12.4 (10.4–14.4)	442	4.9 (4.5–5.4)	1,139	5.1 (4.8–5.4)	49	14,744	8.0 (7.8–8.1)
Total	42,146	18 (17.9–18.2)	5,743	14.1 (13.7–14.5)	644	25.4 (23.4–27.5)	1,330	8.0 (7.5–8.4)	4,877	10.6 (10.3–10.9)	201	54,941	16.3 (16.1–16.4)
**2015**													
Male	31,353	28.8 (28.5–29.1)	5,007	25.8 (25.1–26.6)	490	39.2 (35.5–42.8)	902	11.2 (10.4–11.9)	3,970	16.8 (16.2–17.4)	166	41,888	26.3 (26.0–26.5)
Female	12,070	9.2 (9.1–9.4)	1,359	6.3 (6.0–6.6)	195	14.8 (12.7–16.9)	478	5.0 (4.6–5.5)	1,203	5.2 (4.9–5.5)	53	15,358	8.2 (8.1–8.3)
Total	43,423	18.6 (18.4–18.8)	6,366	15.4 (15.0–15.8)	685	26.5 (24.5–28.5)	1,380	7.8 (7.4–8.2)	5,173	10.8 (10.5–11.1)	219	57,246	16.8 (16.6–16.9)
**2016**													
Male	32,241	29.4 (29.1–29.8)	5,359	27.3 (26.5–28.0)	486	38.2 (34.7–41.8)	988	11.6 (10.9–12.3)	4,310	17.5 (16.9–18.1)	141	43,525	26.9 (26.6–27.2)
Female	12,501	9.5 (9.4–9.7)	1,498	6.8 (6.5–7.2)	166	12.4 (10.4–14.3)	540	5.3 (4.9–5.8)	1,275	5.2 (4.9–5.5)	29	16,009	8.5 (8.3–8.6)
Total	44,742	19.1 (18.9–19.3)	6,857	16.4 (16.0–16.8)	652	24.8 (22.9–26.8)	1,528	8.2 (7.8–8.6)	5,585	11.2 (10.9–11.5)	170	59,534	17.3 (17.1–17.4)
**2017**													
Male	33,209	30.0 (29.6–30.3)	5,577	27.8 (27.0–28.5)	542	42.2 (38.5–45.9)	1,041	11.9 (11.1–12.6)	4,463	17.9 (17.3–18.4)	129	44,961	27.4 (27.2–27.7)
Female	12,688	9.6 (9.4–9.8)	1,473	6.6 (6.3–7.0)	210	15.5 (13.3–17.6)	512	4.8 (4.4–5.3)	1,254	5.1 (4.8–5.4)	33	16,170	8.4 (8.3–8.6)
Total	45,897	19.4 (19.2–19.6)	7,050	16.6 (16.2–17.0)	752	28.3 (26.2–30.4)	1,553	8.0 (7.6–8.4)	5,717	11.3 (10.9–11.6)	162	61,131	17.5 (17.3–17.6)

**Abbreviation:** CI = confidence interval.

* Death estimates obtained from CDC’s National Vital Statistics System. Visits with missing age or sex were excluded; numbers subject to rounding error.

^†^ Per 100,000 population, age-adjusted to the 2000 U.S. standard population, using 12 age groups: 0–4, 5–9, 10–14, 15–19, 20–24, 25–34, 35–44, 45–54, 55–64, 65–74, 74–84, and ≥85 years.

^§^ Record-axis condition codes were used (usually included both part I and part II of entity-axis condition codes). https://www.cdc.gov/nchs/data/datalinkage/underlying_and_multiple_causes_of_death557_2011.pdf.

^¶^ Persons who were white, black, American Indian/Alaska Native, Asian/ Pacific Islander, or Other were non-Hispanic; Hispanics could be of any race.

** Differences in any two rates were considered statistically significant if their CIs were not overlapping.

Unintentional TBIs combined across mechanism of injury were responsible for a higher number and rate of deaths than were suicide and homicide across all study years (p<0.001) (Table 2). Unintentional motor vehicle crashes led to the highest number and rate of all TBI-related deaths from 2000–2002 to 2006–2008 (p<0.05). Beginning in 2009–2011 and continuing through 2015–2017, suicide was responsible for the most TBI-related deaths (p<0.001). Across all data years, firearm-related injuries were responsible for approximately 97% of all TBI-related suicides. The leading category of TBI-related injury death varied by race/ethnicity and changed for some groups during the study period. For example, from 2000–2002 to 2003–2005, unintentional motor vehicle crashes accounted for the highest rate of TBI-related deaths for whites (p<0.001). Beginning in 2006–2008 and continuing through 2015–2017, suicide accounted for the highest rate of TBI-related deaths for this group (p<0.002). Among blacks, homicide was responsible for the highest rate of TBI-related deaths from 2000–2002 to 2015–2017 (p<0.001). Across the study period, the highest rate of TBI-related deaths among AI/AN was attributed to unintentional motor vehicle crashes (p<0.05). Among Hispanics, unintentional motor vehicle crashes were the most common cause of TBI-related deaths from 2000–2002 to 2006–2008 (p<0.001). During 2009–2011, the rates of TBI-related death from unintentional motor vehicle crashes and unintentional falls were similar (p = 0.16) in Hispanics; beginning in 2012–2014 and through 2015–2017, unintentional falls were the most common cause of TBI-related deaths among Hispanics (p<0.001).

**TABLE 2:** Estimated average annual number* and age-adjusted rates^†^ per 100,000 population of traumatic brain injury (TBI)–related deaths^§^ by year, intent, mechanism of injury, and race/ethnicity^¶^ — United States, 2007–2017**

3-year interval/mechanism of injury	Race/Ethnicity											Total	
	White		Black		American Indian/Alaska Native		Asian/Pacific Islander		Hispanic		Other		
	No.	Rate (95% CI)	No.	Rate (95% CI)	No.	Rate (95% CI)	No.	Rate (95% CI)	No.	Rate (95% CI)	No.	No.	Rate (95% CI)
**2000–2002**													
**Total unintentional TBI-related deaths**	**22,908**	**11.0 (10.9–11.1)**	**2,914**	**8.9 (8.7–9.1)**	**414**	**18.5 (17.4–19.6)**	**622**	**6.8 (6.5–7.2)**	**2,940**	**9.3 (9.1–9.6)**	**103**	**29,902**	**10.5 (10.4–10.6)**
Unintentional motor vehicle crashes	12,416	6.3 (6.2–6.4)	1,919	5.4 (5.2–5.5)	311	12.8 (12.0–13.7)	343	3.0 (2.8–3.2)	2,014	5.3 (5.1–5.4)	52	17,055	6.0 (5.9–6.0)
Unintentional falls^††^	6,496	2.8 (2.8–2.8)	477	1.8 (1.7–1.9)	53	3.4 (2.8–4.0)	194	2.8 (2.6–3.1)	484	2.5 (2.4–2.7)	30	7,734	2.7 (2.7–2.8)
Unintentionally struck by/against an object	304	0.1 (0.1–0.2)	34	0.1 (0.1–0.1)	2^§§^	0.1(0.0–0.2)	5^§§^	0.0 (0.0–0.1)^§§^	46	0.1 (0.1–0.2)	2^§§^	393	0.1 (0.1–0.1)
Other unintentional injury, mechanism unspecified^¶¶^	3,692	1.7 (1.7–1.8)	484	1.6 (1.5–1.7)	48	2.2 (1.8–2.6)	81	0.9 (0.8–1.1)	396	1.4 (1.3–1.5)	19^§§^	4,719	1.7 (1.6–1.7)
**Total intentional TBI-related deaths**	**14,312**	**6.9 (6.9–7.0)**	**3,258**	**9.0 (8.8–9.1)**	**188**	**7.9 (7.3–8.6)**	**307**	**2.6 (2.4–2.7)**	**1,718**	**4.8 (4.6–4.9)**	**98**	**19,882**	**7.0 (6.9–7.0)**
Suicide	11,909	5.7 (5.7–5.8)	883	2.5 (2.4–2.6)	102	4.4 (3.9–4.9)	164	1.4 (1.2–1.5)	728	2.3 (2.2–2.4)	46	13,833	4.8 (4.8–4.9)
Homicide	2,403	1.2 (1.2–1.2)	2,375	6.4 (6.3–6.6)	86	3.6 (3.1–4.0)	143	1.2 (1.1–1.3)	990	2.5 (2.4–2.6)	51	6,049	2.1 (2.1–2.1)
Other (no intent or mechanism specified)***	348	0.2 (0.2–0.2)	96	0.3 (0.3–0.3)	13^§§^	0.6 (0.4–0.8)^§§^	14^§§^	0.1 (0.1–0.2)^§§^	79	0.2 (0.2–0.3)	9^§§^	559	0.2 (0.2–0.2)
**Total**	**37,568**	**18.1 (18.0–18.2)**	**6,269**	**18.2 (17.9–18.4)**	**615**	**27.0 (25.7–28.3)**	**943**	**9.5 (9.1–9.9)**	**4,737**	**14.3 (14.1–14.6)**	**210**	**50,342**	**17.6 (17.6–17.7)**
**2003–2005**													
**Total unintentional TBI-related deaths**	**23,940**	**11.1 (11.0–11.2)**	**3,009**	**9.0 (8.8–9.2)**	**406**	**17.5 (16.4–18.5)**	**709**	**6.9 (6.6–7.2)**	**3,181**	**9.3 (9.0–9.5)**	**81**	**31,326**	**10.6 (10.5–10.7)**
Unintentional motor vehicle crashes	11,827	5.9 (5.9–6.0)	1,873	5.1 (5.0–5.3)	276	10.8 (10.0–11.5)	349	2.8 (2.6–2.9)	2,146	5.1 (5.0–5.2)	46	16,516	5.6 (5.6–5.7)
Unintentional falls^††^	8,325	3.4 (3.4–3.5)	570	2.1 (2.0–2.2)	65	3.8 (3.2–4.4)	272	3.3 (3.0–3.5)	609	3.0 (2.8–3.1)	22	9,863	3.3 (3.3–3.4)
Unintentionally struck by/against an object	286	0.1 (0.1–0.1)	33	0.1 (0.1–0.1)	5^§§^	0.2(0.1–0.4)^§§^	7	0.1 (0.0–0.1)^§§^	50	0.1 (0.1–0.2)	1^§§^	381	0.1 (0.1–0.1)
Other unintentional injury, mechanism unspecified^¶¶^	3,502	1.6 (1.6–1.7)	534	1.7 (1.6–1.8)	61	2.7 (2.3–3.1)	82	0.8 (0.7–0.9)	376	1.1 (1.0–1.1)	13^§§^	4,566	1.5 (1.5–1.6)
**Total intentional TBI-related deaths**	**14,482**	**6.9 (6.8–6.9)**	**3,286**	**8.7 (8.5–8.9)**	**205**	**8.2 (7.6–8.9)**	**302**	**2.2 (2.1–2.4)**	**1,823**	**4.5 (4.3–4.6)**	**69**	**20,168**	**6.8 (6.8–6.9)**
Suicide	12,305	5.8 (5.7–5.8)	851	2.4 (2.3–2.4)	112	4.6 (4.1–5.1)	168	1.3 (1.1–1.4)	754	2.1 (2.0–2.1)	36	14,225	4.8 (4.8–4.8)
Homicide	2,177	1.1 (1.1–1.1)	2,436	6.3 (6.2–6.5)	93	3.6 (3.2–4.1)	135	1.0 (0.9–1.1)	1,069	2.4 (2.3–2.5)	34	5,943	2.0 (2.0–2.0)
Other (no intent or mechanism specified)***	382	0.2 (0.2–0.2)	135	0.4 (0.3–0.4)	15^§§^	0.6 (0.5–0.9)^§§^	12	0.1 (0.1–0.1)^§§^	95	0.2 (0.2–0.3)	4^§§^	643	0.2 (0.2–0.2)
**Total**	**38,804**	**18.2 (18.1–18.3)**	**6,430**	**18.1 (17.8–18.3)**	**626**	**26.3 (25.1–27.6)**	**1,024**	**9.2 (8.9–9.6)**	**5,098**	**14.0 (13.7–14.2)**	**154**	**52,137**	**17.6 (17.5–17.7)**
**2006–2008**													
**Total unintentional TBI-related deaths**	**24,156**	**10.8 (10.7–10.9)**	**2,829**	**8.1 (7.9–8.2)**	**372**	**15.4 (14.4–16.3)**	**750**	**6.4 (6.2–6.7)**	**3,133**	**8.4 (8.2–8.6)**	**68**	**31,308**	**10.1 (10.1–10.2)**
Unintentional motor vehicle crashes	10,662	5.3 (5.2–5.3)	1,724	4.5 (4.4–4.6)	243	9.2 (8.5–9.9)	329	2.4 (2.3–2.6)	1,952	4.2 (4.1–4.3)	33	14,943	4.9 (4.9–5.0)
Unintentional falls^††^	9,920	3.9 (3.9–3.9)	591	2.1 (2.0–2.2)	74	4.0 (3.4–4.5)	345	3.4 (3.2–3.6)	741	3.1 (2.9–3.2)	23	11,694	3.7 (3.7–3.8)
Unintentionally struck by/against an object	283	0.1 (0.1–0.1)	33	0.1 (0.1–0.1)	3^§§^	0.1 (0.1–0.2)^§§^	7	0.1 (0.0–0.1)^§§^	55	0.1 (0.1–0.1)	0^§§^	381	0.1 (0.1–0.1)
Other unintentional injury, mechanism unspecified^¶¶^	3,291	1.5 (1.5–1.5)	481	1.4 (1.3–1.5)	52	2.1 (1.8–2.5)	70	0.6 (0.5–0.7)	385	1.0 (0.9–1.1)	12^§§^	4,290	1.4 (1.4–1.4)
**Total intentional TBI-related deaths**	**15,125**	**7.0 (7.0–7.1)**	**3,339**	**8.4 (8.3–8.6)**	**189**	**7.3 (6.7–7.9)**	**301**	**2.1 (1.9–2.2)**	**1,844**	**4.2 (4.0–4.3)**	**55**	**20,854**	**6.8 (6.8–6.9)**
Suicide	12,913	5.9 (5.9–6.0)	876	2.3 (2.2–2.4)	107	4.2 (3.7–4.6)	176	1.2 (1.1–1.3)	795	2.0 (1.9–2.1)	36	14,903	4.8 (4.8–4.9)
Homicide	2,212	1.1 (1.1–1.1)	2,463	6.1 (6.0–6.3)	82	3.1 (2.7–3.5)	125	0.9 (0.8–1.0)	1,049	2.2 (2.1–2.2)	20^§§^	5,950	2.0 (1.9–2.0)
Other (no intent or mechanism specified)***	422	0.2 (0.2–0.2)	134	0.4 (0.3–0.4)	18^§§^	0.8 (0.6–1.0)^§§^	20	0.1 (0.1–0.2)^§§^	108	0.3 (0.2–0.3)	4^§§^	706	0.2 (0.2–0.2)
**Total**	**39,704**	**18.0 (17.9–18.2)**	**6,301**	**16.9 (16.6–17.1)**	**579**	**23.4 (22.3–24.6)**	**1,071**	**8.7 (8.4–9.0)**	**5,085**	**12.9 (12.6–13.1)**	**128**	**52,867**	**17.2 (17.1–17.3)**
**2009–2011**													
**Total unintentional TBI-related deaths**	**22,629**	**9.7 (9.6–9.8)**	**2,444**	**6.7 (6.6–6.9)**	**347**	**14.7 (13.7–15.6)**	**773**	**6.0 (5.7–6.2)**	**2,695**	**7.0 (6.8–7.1)**	**81**	**28,969**	**9.0 (8.9–9.1)**
Unintentional motor vehicle crashes	8,112	4.0 (3.9–4.0)	1,365	3.4 (3.3–3.5)	202	7.6 (7.0–8.2)	270	1.7 (1.6–1.9)	1,498	3.0 (2.9–3.1)	35	11,482	3.7 (3.6–3.7)
Unintentional falls^††^	11,281	4.3 (4.2–4.3)	675	2.2 (2.1–2.3)	87	4.7 (4.1–5.3)	415	3.6 (3.4–3.8)	810	3.1 (3.0–3.2)	32	13,301	4.0 (4.0–4.0)
Unintentionally struck by/against an object	276	0.1 (0.1–0.1)	31	0.1 (0.1–0.1)	2^§§^	0.1 (0.0–0.2)^§§^	8	0.1 (0.0–0.1)^§§^	43	0.1 (0.1–0.1)	0^§§^	360	0.1 (0.1–0.1)
Other unintentional injury, mechanism unspecified^¶¶^	2,960	1.3 (1.3–1.4)	373	1.0 (1.0–1.1)	56	2.2 (1.9–2.6)	80	0.6 (0.5–0.6)	343	0.8 (0.8–0.9)	14^§§^	3,826	1.2 (1.2–1.2)
**Total intentional TBI-related deaths**	**16,465**	**7.6 (7.5–7.7)**	**3,016**	**7.4 (7.3–7.6)**	**216**	**8.3 (7.6–8.9)**	**321**	**2.0 (1.8–2.1)**	**1,768**	**3.7 (3.6–3.8)**	**90**	**21,877**	**6.9 (6.9–7.0)**
Suicide	14,416	6.6 (6.5–6.7)	908	2.3 (2.2–2.4)	130	5.0 (4.5–5.5)	204	1.2 (1.1–1.3)	867	2.0 (1.9–2.0)	55	16,580	5.2 (5.1–5.2)
Homicide	2,049	1.0 (1.0–1.0)	2,109	5.1 (5.0–5.2)	86	3.3 (2.9–3.7)	117	0.7 (0.6–0.8)	901	1.7 (1.6–1.8)	35	5,297	1.7 (1.7–1.7)
Other (no intent or mechanism specified)***	448	0.2 (0.2–0.2)	130	0.3 (0.3–0.4)	23	0.9 (0.7–1.1)	16^§§^	0.1 (0.1–0.1)^§§^	103	0.2 (0.2–0.3)	6^§§^	726	0.2 (0.2–0.2)
**Total**	**39,542**	**17.5 (17.4–17.6)**	**5,591**	**14.5 (14.3–14.7)**	**586**	**23.9 (22.7–25.0)**	**1,110**	**8.0 (7.7–8.3)**	**4,566**	**10.9 (10.7–11.1)**	**177**	**51,572**	**16.1 (16.0–16.2)**
**2012–2014**													
**Total unintentional TBI-related deaths**	**23,486**	**9.7 (9.6–9.8)**	**2,530**	**6.6 (6.5–6.8)**	**373**	**15.2 (14.2–16.1)**	**888**	**5.9 (5.7–6.2)**	**2,895**	**7.0 (6.9–7.2)**	**87**	**30,260**	**9.0 (8.9–9.0)**
Unintentional motor vehicle crashes	7,566	3.7 (3.7–3.8)	1,370	3.3 (3.2–3.4)	212	7.8 (7.2–8.4)	257	1.5 (1.4–1.6)	1,482	2.8 (2.7–2.8)	29	10,916	3.4 (3.4–3.4)
Unintentional falls^††^	12,677	4.6 (4.5–4.6)	746	2.2 (2.1–2.3)	104	5.1 (4.5–5.7)	527	3.8 (3.6–4.0)	1,006	3.4 (3.2–3.5)	46	15,107	4.3 (4.2–4.3)
Unintentionally struck by/against an object	276	0.1 (0.1–0.1)	28	0.1 (0.1–0.1)	4^§§^	0.2 (0.1–0.3)^§§^	10	0.1 (0.0–0.1)^§§^	48	0.1 (0.1–0.1)	0^§§^	367	0.1 (0.1–0.1)
Other unintentional injury, mechanism unspecified^¶¶^	2,967	1.3 (1.3–1.3)	386	1.0 (0.9–1.1)	53	2.1 (1.8–2.4)	94	0.6 (0.5–0.6)	359	0.8 (0.7–0.8)	11^§§^	3,870	1.2 (1.1–1.2)
**Total intentional TBI-related deaths**	**17,692**	**8.1 (8.0–8.2)**	**3,100**	**7.4 (7.2–7.5)**	**239**	**9.2 (8.5–9.9)**	**363**	**2.0 (1.8–2.1)**	**1,750**	**3.4 (3.3–3.5)**	**83**	**23,227**	**7.1 (7.1–7.2)**
Suicide	15,755	7.1 (7.1–7.2)	966	2.4 (2.3–2.5)	155	6.0 (5.4–6.5)	242	1.3 (1.2–1.4)	959	1.9 (1.9–2.0)	60	18,138	5.5 (5.4–5.5)
Homicide	1,937	1.0 (0.9–1.0)	2,134	5.0 (4.9–5.1)	84	3.2 (2.8–3.6)	121	0.7 (0.6–0.7)	791	1.4 (1.4–1.5)	23	5,089	1.6 (1.6–1.6)
Other (no intent or mechanism specified)***	465	0.2 (0.2–0.2)	123	0.3 (0.3–0.3)	23	0.9 (0.7–1.1)	20	0.1 (0.1–0.1)	112	0.2 (0.2–0.3)	7^§§^	751	0.2 (0.2–0.2)
**Total**	**41,644**	**18.0 (17.9–18.1)**	**5,754**	**14.3 (14.1–14.5)**	**635**	**25.2 (24.0–26.4)**	**1,271**	**8.0 (7.7–8.3)**	**4,757**	**10.6 (10.4–10.8)**	**177**	**54,238**	**16.3 (16.2–16.4)**
**2015–2017**													
**Total unintentional TBI-related deaths**	**24,843**	**9.9 (9.8–10.0)**	**2,919**	**7.3 (7.1–7.4)**	**391**	**15.3 (14.4–16.2)**	**1,018**	**5.7 (5.5–5.9)**	**3,273**	**7.2 (7.0–7.3)**	**102**	**32,547**	**9.2 (9.1–9.2)**
Unintentional motor vehicle crashes	7,508	3.7 (3.6–3.7)	1,579	3.7 (3.6–3.8)	211	7.7 (7.1–8.3)	279	1.4 (1.3–1.5)	1,627	2.8 (2.8–2.9)	32	11,236	3.4 (3.4–3.5)
Unintentional falls^††^	13,977	4.8 (4.8–4.9)	859	2.4 (2.3–2.5)	109	4.8 (4.3–5.3)	626	3.7 (3.6–3.9)	1,203	3.4 (3.3–3.6)	54	16,828	4.5 (4.4–4.5)
Unintentionally struck by/against an object	257	0.1 (0.1–0.1)	24	0.1 (0.0–0.1)	4^§§^	0.1 (0.1–0.2)^§§^	6	0.0 (0.0–0.0)^§§^	47	0.1 (0.1–0.1)	1^§§^	339	0.1 (0.1–0.1)
Other unintentional injury, mechanism unspecified^¶¶^	3,100	1.3 (1.3–1.3)	457	1.1 (1.1–1.2)	67	2.6 (2.2–3.0)	108	0.6 (0.5–0.6)	396	0.8 (0.8–0.9)	16^§§^	4,144	1.2 (1.2–1.2)
**Total intentional TBI-related deaths**	**19,367**	**8.9 (8.8–9.0)**	**3,701**	**8.5 (8.4–8.7)**	**275**	**10.1 (9.4–10.9)**	**445**	**2.2 (2.0–2.3)**	**2,101**	**3.7 (3.6–3.8)**	**75**	**25,965**	**7.8 (7.7–7.8)**
Suicide	17,236	7.8 (7.7–7.9)	1,187	2.8 (2.7–2.9)	184	6.9 (6.3–7.5)	323	1.6 (1.5–1.7)	1,200	2.2 (2.1–2.3)	55	20,186	5.9 (5.9–6.0)
Homicide	2,131	1.1 (1.0–1.1)	2,514	5.7 (5.6–5.9)	90	3.3 (2.9–3.7)	122	0.6 (0.5–0.7)	901	1.5 (1.5–1.6)	20	5,779	1.8 (1.8–1.8)
Other (no intent or mechanism specified)***	477	0.2 (0.2–0.2)	137	0.3 (0.3–0.4)	31	1.2 (0.9–1.4)	23	0.1 (0.1–0.1)	118	0.2 (0.2–0.2)	6^§§^	792	0.2 (0.2–0.2)

**Abbreviation:** CI = confidence interval.

* Death estimates obtained from CDC’s National Vital Statistics System. Visits with missing age were excluded; numbers subject to rounding error.

^†^ Per 100,000 population, age-adjusted to the 2000 U.S. standard population, using 12 age groups: 0–4, 5–9, 10–14, 15–19, 20–24, 25–34, 35–44, 45–54, 55–64, 65–74, 74–84, and ≥85 years.

^§^ Record-axis condition codes were used (usually included both part I and part II of entity-axis condition codes). https://www.cdc.gov/nchs/data/datalinkage/underlying_and_multiple_causes_of_death557_2011.pdf.

^¶^ Persons who were white, black, American Indian/Alaska Native, Asian/ Pacific Islander, or Other were non-Hispanic; Hispanics could be of any race.

** Differences in any two rates were considered statistically significant if their CIs were not overlapping.

^††^ Includes falls of undetermined intent to maintain consistency with past data releases.

^§§^ Rates based on ≤20 deaths might be unstable and should be interpreted with caution.

^¶¶^ External cause of injury codes specify that the injury was unintentional but do not specify the actual mechanism of injury.

*** Includes TBIs for which the intent was not determined as well as those caused by legal intervention or war. Includes TBIs in which no mechanism was specified in the record. Does not include falls of undetermined intent.

## Discussion

Over the 18-year study period, approximately 960,000 TBI-related deaths occurred in the United States; however, the patterns differed over time and among racial/ethnic groups. Whereas the rates of TBI-related deaths among whites and blacks were similar from 2001 to 2006, the rates among whites subsequently exceeded those among blacks, presumably related to a 32% increase in TBI-related suicide deaths among whites, from 5.9 per 100,000 during 2006–2008 to 7.8 during 2015–2017. Previous data have documented an increasing prevalence of suicide among whites and AI/ANs (*6*). These findings suggest that tailored prevention efforts might be needed to help reduce the prevalence of TBI among different groups at risk for injury.

This analysis corroborated findings in a 2017 study of TBI-related emergency department visits, hospitalizations, and deaths (*1*) that identified a shift in the leading category of TBI-related deaths in the United States during the last 10 years from unintentional motor vehicle crashes to suicide. That shift was driven by a significant increase in TBI-related suicide deaths as well as an overall decrease in motor vehicle crash deaths during the last decade (*7*). CDC supports suicide prevention efforts by encouraging the use of strategies that reflect the best available evidence, including strengthening access and delivery of suicide care, creating protective environments, teaching coping and problem-solving skills, and identifying and supporting persons at risk (*8*). Firearm injury was the underlying mechanism of injury in nearly all TBI-related suicides among all groups. Reducing access to lethal means among persons at risk for suicide is an important approach to creating protective environments (*8*).

Also consistent with previous research, AI/ANs consistently had the highest age-adjusted rates of TBI-related deaths across the study period, and unintentional motor vehicle crashes contributed the highest number and accounted for the highest rate of these TBI-related deaths in all years (*9*). Lower rates of seat belt use and higher rates of alcohol-related motor vehicle crash deaths among AI/ANs compared with other groups might be contributing factors (*9*). Expansion of evidence-based strategies for reducing the likelihood of injury once a motor vehicle crash has occurred, for example enactment of universal motorcycle helmet laws and enforcement of existing seat belt and child restraint/booster laws, might be beneficial.^§^

TBI-related homicides disproportionately affected blacks compared with all other groups. CDC’s National Center for Injury Prevention and Control has created technical packages that outline the best available evidence-based strategies for preventing violence^¶^; the strategies are intended to work together and to be used in combination in a multilevel, multisector effort to prevent violence. Implementation might help stop violence before it starts and decrease the rates of TBI-related homicides.

Falls are the second-leading cause of TBI-related deaths and have been increasing in number and rate, particularly among older adults (*1*). Health care providers can play an important role in in the prevention of older adult falls. CDC’s STEADI** (Stopping Elderly Accidents Deaths and Injuries) initiative can help providers address patient fall risk through the identification of modifiable risk factors and implementation of effective interventions (e.g., strength and balance exercises and medication management).

The findings in this report are subject to at least two limitations. First, misclassification of race and Hispanic origin is a common problem on death certificates, especially for AI/AN, Asian/PI, and Hispanic populations (*10*). Therefore, for these groups, mortality estimates are most likely underestimates. Second, incomplete reporting or misclassification of cause of death on death certificates might bias estimates of TBI-related deaths.

Understanding the leading contributors to TBI-related death and identifying groups at increased risk is important in preventing this injury. Health care providers can play an important role in assessing patients at increased risk, such as those at risk for suicide, unintentional motor vehicle crashes, or unintentional falls, and provide referrals or tailored interventions.

**Summary**

**What is already known about this topic?**

Traumatic brain injuries (TBIs) contribute to a substantial number of deaths each year.

**What is added by this report?**

In 2017, approximately 61,000 TBI-related deaths occurred in the United States. Suicide surpassed motor vehicle crashes as the leading category of TBI-related deaths during 2009–2011 and through 2015–2017. Males and American Indians/Alaska Natives experienced the highest rates of TBI-related death.

**What are the implications for public health practice?**

Broader implementation of evidence-based prevention strategies for the leading categories of TBI-related death, particularly those aimed at stemming the significant increase in suicide, are warranted. Health care providers can play an important role in assessing patients at increased risk for suicide and providing appropriate interventions.

## Footnotes

*
https://www.cdc.gov/nchs/products/databriefs/db328.htm .

† Previous estimates of TBI-related deaths included all cases in which a TBI-related ICD-10 code was listed in the NVSS mortality record, regardless of whether an injury code was listed as the underlying cause of death. The current methodology only includes deaths for which an injury was more directly related to the cause of death.

§ https://www.cdc.gov/motorvehiclesafety/index.html.

¶ https://www.cdc.gov/violenceprevention/pub/technical-packages.html.

** https://www.cdc.gov/steadi/index.html.
